# The Effect of Simulation Nursing Education Using the Outcome-Present State-Test Model on Clinical Reasoning, the Problem-Solving Process, Self-Efficacy, and Clinical Competency in Korean Nursing Students

**DOI:** 10.3390/healthcare9030243

**Published:** 2021-02-24

**Authors:** Yon Hee Seo, Mi Ran Eom

**Affiliations:** 1 Department of Nursing, Yeoju Institute of Technology, Yeoju 12652, Gyeonggido, Korea; yseo017@naver.com; 2 Department of Nursing, Mokpo National University, Muan-gun 58544, Jeollanamdo, Korea

**Keywords:** clinical competency, clinical reasoning, Outcome-Present State-Test model, problem solving process, self-efficacy, simulation nursing education program

## Abstract

The purpose of this study was to assess the effect of a simulation nursing education program in terms of clinical reasoning, problem-solving process, self-efficacy, and clinical competency using the Outcome-Present State-Test (OPT) model in nursing students. The participants comprised 45 undergraduate nursing students recruited from two universities in Korea. The number of nursing students assigned to the experimental group and control group were 25 and 20, respectively. For a period of two weeks, the experimental group received a simulation nursing education program using the OPT model, while the control group received a traditional clinical practicum. The data were analyzed using prior homogeneity tests (Fisher’s exact test and paired *t*-test); ANCOVA was performed to investigate the differences in dependent variables between the two groups. There was a significant improvement in clinical reasoning (F = 10.59, *p* = 0.002), problem-solving process (F = 30.92, *p* < 0.001), and self-efficacy (F = 36.03, *p* < 0.001) in the experimental group as compared to the control group (F = 10.59, *p* = 0.002). Moreover, the experimental group showed significantly higher scores in clinical competency than the control group (F = 11.07, *p* = 0.002). This study demonstrates that the simulation nursing education program using the OPT model for undergraduate students is very effective in promoting clinical reasoning, problem-solving processes, self-efficacy, and clinical competency.

## 1. Introduction

Medical education and healthcare institutions have been facing several problems because of the COVID-19 pandemic, such as halting field placements, considering the safety of students. Recently, educational institutions have designed various alternative plans for practical training, such as virtual reality (VR) and augmented reality (AR), and solutions such as Mosby’s Nursing Skills (Elsevier) [1,2].

Practical simulation training is a teaching method to help students gain nursing knowledge and skills through debriefing after reproducing clinical scenarios using a simulator [1]. It involves materializing a clinical environment that is similar to actual conditions, thereby facilitating nursing practice in a safe, virtual environment; currently, alternative practical training using simulation is being widely used across nursing schools, as field placements have been disrupted due to the pandemic [2]. In general, practical simulation training is known to improve nursing skills among nursing school students in addition to improving their confidence, satisfaction, anxiety, stress, and academic motivation [3,4,5]. Despite these advantages, nursing school students may be unable to apply in practice the skills they acquire in practical simulation training [6].

Clinical reasoning, which is one of the most important core skills required in nursing, is a cognitive process that involves examining and analyzing clues associated with patients to formulate a nursing plan and achieve desirable results [7]; in fact, it is regarded that the quality of nursing is determined by the clinical reasoning skills of nurses [8]. However, most studies in the process of clinical reasoning are limited because they are an analysis of self-reported questionnaires [9], indicating a lack of studies using rubrics to objectively measure the clinical reasoning skills of nursing school students.

Problem-solving ability is the ability to handle a problematic situation that occurs in daily life, and it focuses on the process rather than the result of behavior. This problem-solving process is an important factor that nurses should acquire, as it can help in decision making for an effective knowledge-based problem-solving strategy [10], and help build clinical reasoning and judgment needed to solve nursing problems [11,12]. Additionally, nurses should acquire a rapid and clear problem-solving ability in the nursing field and clinical competency to deal with various health management demands. These include improving the self-efficacy of nursing students, as higher self-efficacy leads to self-confidence in the ability to perform nursing tasks to solve nursing problems, which can contribute toward increasing the overall quality of nursing [13].

Recently, the Outcome-Present State-Test (OPT) model has been gaining traction as a third-generation nursing process model that provides structure for nursing students’ thought processes [14,15,16] and for conceptualizing the clinical reasoning process [15]. The OPT model consists of outline, framing, client-in-context story, relationship, and theoretical evidence with nursing diagnosis, nursing intervention, and determination of current and expected results for clinical reasoning [15]. Unlike the second-generation nursing process models, this model emphasizes the patient situation and outline [15]. Moreover, it helps improve the problem-solving ability among nurses with respect to patient problems in the present state [17]; it reinforces thinking skills, as students analyze nursing problems from different aspects based on a high-level thinking process using the outline of the OPT model [16,17]. Nevertheless, advanced studies on simulation education using the OPT model have been limited [18,19]. Thus, the purpose of this study is to investigate the effect of simulation in nursing education using the OPT model on students’ clinical reasoning, problem-solving process, self-efficacy, and clinical competency.

## 2. Materials and Methods

### 2.1. Participants

**Figure 1:**
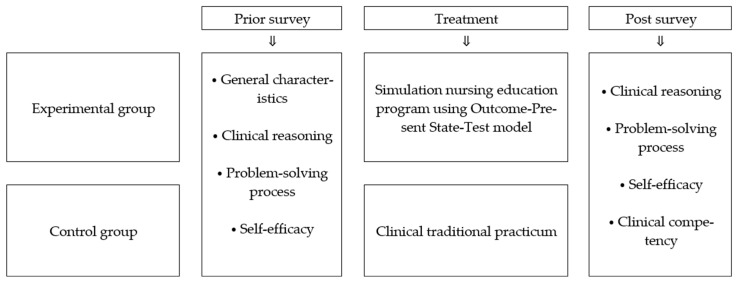
Research design.

The participants of this study were senior-year nursing students of Mokpo National University and Chodang University in Korea, with the following selection criteria: (1) they understand the purpose of this study and provide written consent for participation, (2) they have completed the fundamentals of nursing and health assessment that are within the department of nursing science, (3) they must have completed at least one semester of clinical practicum curriculum, and (4) they have no previous experience with simulation education. This study was conducted after obtaining ethics approval from the Institutional Review Board of Mokpo National University (MNUIRB-20150608-SB-005-02). The sample size of this study was calculated as 20 for each group by setting number of groups = 2 (u = 1), significant level (α) = 0.05, power (1 − β) = 0.70, and effect size (d) = 0.40 as per Cohen’s table [20]. This study recruited a total of 46 subjects—25 in the experimental group and 21 in the control group, considering the drop-out rate; one subject in the control group was excluded for not attending the posttest for individual conditions. Thus, the final number of participants was 45—25 for the experiment group and 20 for the control group. This study used a non-equivalent control group pretest-posttest design to examine the effect of a simulation nursing education program using the OPT model. The design of this study is shown in Figure 1.

### 2.2. Experiment Treatment

The experimental treatment in this study was conducted three times every two weeks from July 13 to 22 August 2015. It comprised the gastrointestinal tract bleeding example from day 1 to 5 and acute myocardial infarction from day 6 to 10 as one set for two weeks to reflect the OPT model well; each education program is shown in Table 1.

Experimental group: Day 1: The instructor provided written feedback of the pre-investigation of clinical reasoning ability by applying gastrointestinal track bleeding, and provided written feedback that was shared with all team members and re-submitted to instructor. The instructor provided the same gastrointestinal track bleeding example to each participant and asked them to identify the nursing problem through self-directed learning using the OPT model work-sheet. The important thing in self-directed learning is to ensure theoretical knowledge about a given example before participating in team discussions, as participating in a team activity or discussion without a pre-class can give some members a free ride or interrupt team cohesion, inhibiting efficient learning [21]. Participants were asked to deduce all clues including inspection result, signs and symptoms, sociality, and family medical history by reviewing the example. During this time, the instructor encouraged the participant to think like a nurse using guideline-based questions after identifying whether the participant could recognize correct and incorrect clues to deduce the nursing problem.

Day 2: A unique characteristic of the OPT model is that it fosters knowledge of the nursing process by deducing actual and potential nursing problems from a subject’s condition and logical clues. Discussion activities for problem solving after acquiring appropriate knowledge is a teaching method that enables individual learning through personal and cooperative learning, iterative learning, continuous feedback, and solving applied questions. Participants were asked to perform learning activities such as sharing and discussing members’ opinions using reference books that were set on each team table and by searching online references. In particular, participants were asked to study the theory to find detailed evidence about signs and symptoms, treatment and nursing, drugs, and so on based on basic conditions of intestinal track bleeding patients. Participants were asked to assess patients and deduce the diagnosis using the clinical reasoning web. Furthermore, participants were asked to present decision-making intervention and testing using an OPT model work-sheet to understand keystone issues and to reach an outcome state from the present state of the nursing subject. In this discussion process, participants were asked to immediately suggest an objection based on the referenced literature, particularly if there was a differing opinion between an individual and team members. In addition, the opposite opinion was accepted if the provided evidence was valid. The instructor played the role of promoter to encourage participants in finding the correct answer through a team discussion without directly suggesting the correct answer. Furthermore, feedback was given that corrected the errors in the problem-solving process, i.e., a wrong answer, during the nursing problem clue recognition process rather than reinforcing the correct answer [22].

Day 3: There should be an assessment purpose and a set priority to ensure exact and systematic clinical reasoning. Thus, the initial stage of clinical practicum was provided to promote efficient transference of theoretically acquired clinical reasoning to the clinical environment. Basic nursing skills were achieved by reflecting nursing intervention and by completing the chosen test in the OPT model work-sheet. Day 3 focused on team activity learning without instruction. It included watching a physical assessment and basic nursing intervention videos regarding the application of knowledge and skills for the physical assessment of a patient. After watching the video, participants moved to the nursing fundamentals skills lab and practiced for 2 h. During this free practice, team members role-played as patients by referring to the objective structured clinical examination (OSCE) guideline that was provided by the researcher for physical assessment practice, and a gastrointestinal track bleeding patient and mannequin were used to practice basic nursing intervention and nursing skill. On Day 3, a self-test quiz was provided by the instructor in the middle of the discussion to check the level of understanding of participants and to encourage active participation by providing a quiz about the learned content.

Day 4: Simulation practice by applying the OPT model to choose and apply basic nursing skill appropriate to specific nursing conditions and priority nursing intervention to solve a patient’s nursing problem. Participants were divided into two teams with four members each, and the instructor provided a scenario, assigning 10 min for brainstorming when one team entered the simulation laboratory. When starting the simulation, a basic electrocardiogram (EKG) wave for patient monitoring and changes such as vital signs and saturation according to the provided nursing intervention and drug delivery was given to allow participants to recognize clues. The instructor provided clues for necessary nursing intervention and clinical judgment. If the participants were not able to recognize the condition change of the patient or were unable to perform the essential nursing intervention in the given time, the grouped moved on to the next stage and the participant was asked to perform the proper assessment test or was orally asked to help the doctor through the reasoning process by recognizing the clues of condition change of the patient during simulation. This reflects the characteristic of the OPT model for clinical judgment, such as identifying changes in the patient’s condition, basic interpretation of inspection results and normal/abnormal result, and re-assessing given nursing intervention effect rather than the action base, such as basic nursing skill performance. Scenario realization time comprised 10 min of gastrointestinal track bleeding and 15 min of acute myocardial infarction. A 1-h debriefing was conducted using a recorded video for each team after finishing the simulation practice. The team that finished simulation practice was asked to write the nursing condition experienced in the simulation practice by using the situation-background-assessment-recommendation (SBAR) patient condition report. Collins [23] reported that using a subject in the safe environment of a simulation can improve skills that are necessary to conduct simulation processes and enhance confidence. Thus, it was combined with a written SBAR to improve communication skills. Participants were asked to submit a reflection paper to the instructor after completing clinical reasoning practice.

Day 5: Conference is a judgment stage of the OPT model, which decides the summary and purpose of the patient’s condition when nursing is finished, and it reflects the debriefing stage that occupies 80% of simulation learning effect to discuss patient’s condition and participant’s behavior [24]. Each team was asked to write the OPT model work-sheet for 2 h through a team discussion by using a similar case simulation video in each independent classroom. This was done to analyze the facts, such as finding the nursing problem-related clue by using indirect experience that solved a similar condition, inspection result, evidence-based nursing for problem solving, and communication between medical team and patient condition report. A conference was conducted for 3 h by two teams with the instructor presenting, followed by a Q&A session and the instructor’s feedback. The instructor promoted efficient clinical reasoning in the conference process by inducing judgment on whether the participant recognized clues related to the nursing problem, what nursing problem was deduced from the given clue, what theoretical evidence was presented regarding the nursing intervention for the present state of the patient, what was the chosen test for re-assessment of provided nursing intervention, and whether there was a change of expected outcome state by intervention effect. Additionally, the instructor encouraged the participant to think and reason effectively by providing immediate feedback about anything that was lacking or wrongly recognized in applying the OPT model.

Day 6–10: Acute MI examples were provided during the 6th to 10th days and the process method was the same as in Days 1–5. In the prior education demand survey, the EKG- and arrhythmia-related pre-class was conducted in a self-direct way as, in the prior education demand survey, nursing students felt cardiovascular disease to be the most difficult, considering the characteristics of the cardiovascular system. On the 10th day, the conference was conducted for 4 h.

The control group participated in adult nursing practice (intensive care unit) for 2 weeks (10 days), equaling 90 h, which is a mandatory class of the Korean nursing department curriculum (Table 1). The control group comprised less than five individuals in each team.

**Figure 2:**
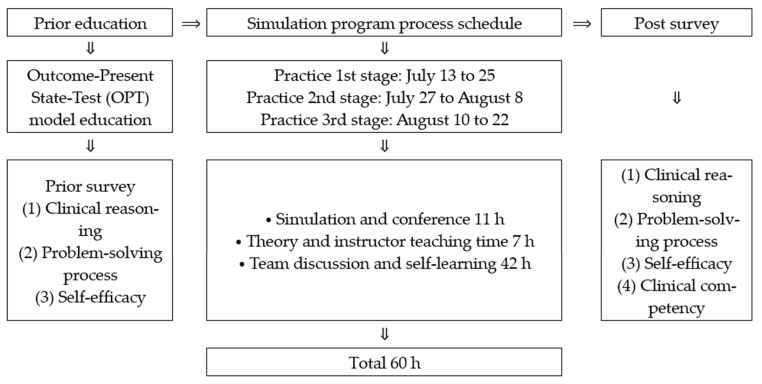
Process of simulation nursing education program using the OPT model.

The education program for the experimental group consisted of 11 h of simulation and conference, including debriefing, seven hours of theory and professor-led instruction, and 42 h of team discussion and self-directed learning at the simulation laboratory at Mokpo National University (Figure 2). The control group participated in clinical training conducted in the intensive care unit of Hankook Hospital in Mokpo city, Korea.

### 2.3. Hypothesis

We propose the following hypothesis: the experimental group who participated in the simulation nursing education program using the OPT model will show better clinical reasoning, problem-solving process, self-efficacy, and clinical competency compared with the control group who participated in clinical traditional practicum.

The hypotheses of this study are as follows: The first hypothesis is that the experimental group that participated in the simulation nursing education program using the OPT model will show improvement in clinical reasoning ability compared with the control group that participated in traditional clinical practicum. The second hypothesis is that the experimental group that participated in the simulation nursing education program using the OPT model will show improvement in the problem-solving process compared with the control group that participated in the traditional clinical practicum. The third hypothesis is that the experimental group that participated in the simulation nursing education program using the OPT model will show improvement in self-efficacy compared with the control group that participated in traditional clinical practicum. The fourth hypothesis is that the experimental group that participated in the simulation nursing education program using the OPT model will show improvement in clinical competency compared with the control group that participated in the traditional clinical practicum.

### 2.4. Research Instruments

The OPT model rating tool developed by Kuiper et al. [18] was used for measuring clinical reasoning. The score range of this instrument is 0 to 78 points, which consists of 20 points for the clinical reasoning web domain and 58 points for the OPT model learning domain, with higher scores indicating a higher ability of clinical reasoning. A Cronbach’s α of 0.88 indicates the reliability of this instrument in this study.

The scale used for quantifying the problem-solving process was developed by Lee [25]. This instrument consists of 25 items, including five items on discovering the problem, five items on defining the problem, five items on designing problem solution, five items on executing the solution to the problem, and five items on examination of the problem-solving process. Each item was measured using a Likert scale that ranged from 1 “absolutely not” to 5 “almost always,” and the score ranged from 25 to 125, with a higher score denoting a higher problem-solving ability. The reliability of the equipment used in this study was estimated using Cronbach’s α, which was 0.96.

To measure self-efficacy, the study used the Neuroscience Nursing Self-Efficacy Scale developed by Dilorio and Price [26]. This instrument comprises 17 items that employs a Likert scale from 0 (“absolutely cannot do”) to 10, (“absolutely can do it”), and the scores range from 0 to 170, with higher scores indicating higher self-efficacy. The reliability of the scale used in this study is conveyed by Cronbach’s α = 0.91.

The Creighton Competency Evaluation Instrument developed by Todd et al. [27] was used to measure clinical competency. It comprised 18 items, including three items on “reasoning,” three items on “communication,” seven items on “clinical decision,” four items on “patient safety,” and one item on “skill level.” The 10 items on reasoning, communication, and safety were scored from 0 (“non-fulfillment”) to 2 points (“complete fulfillment”). Two items of the clinical decision domain—vital sign result interpretation and nursing intervention priority—were measured from 0 (“non-fulfillment”) to 4 points (“complete fulfillment”) by considering the number of fulfillments. Four items, including examination result interpretation, subjective and objective data recognition, suggestion of theoretical evidence of nursing intervention, and evaluation of nursing intervention, were measured from 0 (“non-fulfillment”) to 2 points (“complete fulfillment”). Pain condition was measured on a four-point scale. The score range was 0–42 points, which consisted of the form of an observer evaluation checklist, with a higher score indicating higher clinical competency. The reliability of this study was conveyed by Cronbach’s α = 0.93.

### 2.5. Statistical Analysis

All data from this study are presented as mean ± standard deviations, which were calculated using Windows SPSS (version 21.0; IBM Corp., Armonk, NY, USA). General characteristics of the experimental and control groups were compared, and the prior homogeneity test between the two groups was conducted using Fisher’s exact test, and paired *t*-test. The difference in the intervention effect of the dependent variables of the experimental and control groups was analyzed using analysis of covariance (ANCOVA). Cronbach’s α was used to measure the reliability of the evaluation tool. Statistical significance was set at *p* < 0.05.

## 3. Results

### 3.1. Prior Homogeneity Test

#### 3.1.1. Homogeneity Test for General Characteristics of Subjects

Homogeneity tests according to the general characteristics of the experimental and control groups are presented in Table 2. The subjects of this study were nursing school senior students, and homogeneity was identified as there was no significant difference in gender, satisfaction with clinical practicum, satisfaction with nursing, and satisfaction with college between the experimental and control groups (*p* > 0.05). However, the grade point average of clinical practicum showed a significant difference, and homogeneity was not found (*p* = 0.027).

#### 3.1.2. Homogeneity Test of the Dependent Variables

The prior homogeneity test of the dependent variables is shown in Table 3. Among the dependent variables, clinical reasoning and problem-solving process showed homogeneity between groups (*p* > 0.05), but self-efficacy showed no homogeneity (*p* = 0.016).

### 3.2. Effectiveness Verification

The hypothesis was analyzed using ANCOVA after controlling for covariates, as there was a difference in the grade point average of clinical practicum between groups in the prior homogeneity test.

The clinical reasoning variable showed a significant difference (F = 10.59, *p* = 0.002), as the grade point average of the clinical practicum was not significant as a covariate (F = 0.77, *p* = 0.387), and the clinical reasoning score without adjusting for covariates was 32.04 ± 16.53 in the experimental group and 18.30 ± 12.87 in the control group (Table 4).

The problem-solving process showed a significant difference (F = 30.92, *p* < 0.001), as the grade point average of clinical practicum was not significant as a covariate (F = 1.87, *p* = 0.179), and the problem-solving process score without adjusting for covariates was 99.92 ± 11.42 in the experimental group and 76.65 ± 17.54 in the control group (Table 5).

The self-efficacy variable showed significant difference (F = 36.03, *p* < 0.001) as prior self-efficacy score (F = 19.99, *p* < 0.001) and grade point average of clinical practicum was significant as covariates (F = 5.39, *p* = 0.025); the self-efficacy score without adjusting for covariates was 137.40 ± 22.24 in experimental group and 112.45 ± 27.15 in control group (Table 6).

Clinical competency showed a significant difference (F = 11.07, *p* = 0.002), as the grade point average of clinical practicum was not significant as a covariate (F = 0.50, *p* = 0.484), and the clinical competency score without adjusting for covariates was 31.88 ± 4.46 in the experimental group and 18.90 ± 7.15 in the control group (Table 7).

## 4. Discussion

This study examines whether clinical reasoning, problem-solving processes, self-efficacy, and clinical competency are improved on account of the simulation nursing education program using the OPT model.

First, the experimental group, which underwent the simulation nursing education program using OPT model, showed a significantly higher clinical reasoning score compared with the control group. This result is consistent with the results of a previous study that conducted a 10-week education program using the OPT model among 23 nursing school students who participated in clinical training [28], and a previous study that conducted a four-week education program using the OPT model among 43 nursing school students during clinical training [29]. The OPT model focuses on how the current condition of a subject (present state) changes to the expected result condition (outcome state) and it provides an OPT model outline to improve the clinical reasoning of nursing school students [18]. As a result of conducting simulation education using the OPT model, there was a positive effect on the clinical reasoning of students, as students were able to progress each stage of the OPT model logically through schematization of the flow of systemic thinking about nursing problems, enabling students to identify the situation themselves.

Second, the experimental group showed a significantly higher problem-solving process score compared with the control group. The OPT model focuses on nursing results and is based on metarecognition thinking [29]. Metarecognition is a strategy to adequately apply acquired knowledge to problem-solving by recognizing and adjusting its thinking process [30]. The OPT model, according to Reising [31], which recognizes its thinking process and plan itself, is improved by the checks and evaluations of metarecognition. Thus, when nursing school students perform subject assessment and nursing intervention using the OPT model, they can perform reflective critical thinking to improve the current condition of the subject, promoting metarecognition. Metarecognition understands and controls its own thinking process; therefore, it is assumed that it has a positive effect on improving problem-solving among nursing school students.

Third, the experimental group showed significantly higher self-efficacy compared with the control group. This is consistent with the results of an advanced study that applied the OPT model in the debriefing stage of simulation education [18], and the results of another study that was based on the OPT model worksheet content and self-regulation learning [16]. This appears to be a result of the fact that nursing school students could perform backward thinking to solve nursing problems of the subject, resulting in improvement of critical thinking [31] and creative clinical reasoning. This is linked with the improvement of self-efficacy, as the student can easily prioritize complicated nursing requirements and make decisions on nursing interventions.

Fourth, providing feedback is an important factor for learning; in particular, correction feedback has a positive effect on the promotion of self-efficacy [22,32]. In the feedback method in this study, individual written feedback on clinical reasoning evaluation using the OPT model worksheet was provided to share any constructive feedback, such as accepting a counterpart’s opinion when suggesting definite theoretical evidence as valid if opinions were different. This feedback is not only an essential process to improve the learning ability of nursing school students, but also plays a very important role in promoting self-efficacy [33]. In addition, it is thought that self-efficacy was promoted by the feedback process through the instructor, who induces students to think from other points of view and provides additional information on wrong answers rather than simply stating the right or wrong answers in team discussions and presentations. It follows that undergoing a simulation nursing education program using the OPT model improves the problem-solving process, promoting self-efficacy.

Fifth, the experimental group showed significantly higher clinical competency scores than the control group. This is consistent with previous advanced studies [34]. This study organized a theory learning process using the OPT model in parallel with the simulation field training process. The nursing school students were able to recognize and experience the clue according to changes in patient condition by applying the theoretical knowledge acquired in the classroom during field training. Here, it is assumed that students trained the reasoning process to grasp the exact nursing problem and to solve problems related to various patient conditions. Clinical competency was improved by considering the priority of nursing intervention using a clinical reasoning web. In addition, it is thought that improving clinical reasoning web increases nurses’ confidence in being able to perform nursing activities [23]; moreover, it has a positive effect on clinical competency by practicing summarizing simulation situations using the situation-background-assessment-recommendation style and handing over to colleagues.

Clinical competency in this study was a clinical judgment concept that enabled the selection of a proper nursing intervention as per patient condition priorities, examination result interpretation, drug treatment effect evaluation, and so on, according to the situational nursing problems. Evaluation of clinical competence helped in reinforcing the lack of alternative ability arising from basic repeated skill training. In addition, observed estimation using clinical competency rubric was conducted to measure clinical competency objectively, which was different from advanced studies, such as those using a checklist of core nursing skills and fundamental nursing skills that were suggested by the Korean Accreditation Board of Nursing Education or self-reported questionnaires [35]. The results indicate that the clinical competency of nursing school students was improved by undergoing the OPT model to grasp the overall clinical context and design intervention of nursing problems from various aspects, rather than simply practicing core nursing skills.

This study had some limitations. First, the experimental treatment was conducted over a short period of two weeks, since a previous study reported that the effectiveness of the OPT model was shown in two weeks [28]. It was also conducted as two credits, 60 h, and 10 times for two weeks based on the clinical training credits and rules of the Korean Accreditation Board of Nursing Education. Thus, the program may not be generalizable, as various countries can have different education curriculum and training management. Second, this is the first study that integrated the OPT model and simulation training education; therefore, it has the limitation of determining whether the result is due to the improvement of knowledge by short-term intensive discussion or learning, or due to learning induced by metarecognition through changes in thinking processes, such as backward thinking, obtained from the OPT model learning. Thus, future studies should compare individual learning groups, team learning groups, and clinical training learning groups to clearly identify the effect of applying the OPT model on changes in the thinking processes of nursing school students. Third, it has a limitation of determining whether the result is due to improvement of knowledge by short-term intensive discussion or learning, or due to learning induced by metarecognition through changes in thinking processes, such as backward thinking, obtained from the OPT model learning. Fourth, it is difficult to generalize the results to all Korean nursing school students, as the sample size is small and the participants were from only two university nursing schools in Korea.

This study differed from existing face-to-face education with a simulator, and was a design strategy to deduce the optimal learning effect with a relatively flexible practice method and location by conducting small group team learning and self-directed learning to easily apply a blended learning method of operation that includes minimal face-to-face practice, favoring online practice, expanding both the concept and area. This study is meaningful, as it represents efficient result on the work site by reinforcing the limitations of simulation education suggested by existing advanced studies [6,36,37,38] to achieve the learning goal of relative subject, in a time that clinical training was stopped due to the world facing the COVID-19 pandemic.

## 5. Conclusions

Alternate training plans for nursing school students are an urgent requirement in situations such as the COVID-19 pandemic. The results of this study showed that the simulation nursing education program using the OPT model was effective for improving clinical reasoning, problem-solving process, self-efficacy, and clinical competency in Korean nursing students. Nevertheless, the OPT model in this study may not be generalizable, as various countries may have different education curriculums and training management. For this reason, it is necessary to study the characteristics of each country worldwide, through comparisons and analyses of the nursing education program using the OPT model.

## Figures and Tables

**Table 1:** The content of the simulation nursing education program.

Prior Education			Experimental Group	Control Group
		Outcome-Present State-Test (OPT) Model and Clinical Reasoning Web Education		
Prior Survey		Clinical Reasoning, Problem-Solving Process, Self-Efficacy, Clinical Competency		
Week 1	Theory-oriented clinical reasoning	Day 1	Team organization and building Simulation orientation Deduction of clues related to gastrointestinal track bleeding problem	Working team organization Clinical training department orientation Subject disease condition and grasping severity
		Day 2	Discussion on deduced clue and subject basic condition OPT model work sheet presentation	Subject handover Quiz test Selection of case study with low severity and presentation Deduction of nursing problem clue of case study
	Clinical reasoning training	Day 3	Basic condition video watching Self-quiz test Free practice: objective structured clinical examination (OSCE)	Basic condition of example study subject Open lab (free practice) core skill
		Day 4	Simulation training using OPT model Handover using situation-background-assessment-recommendation (SBAR) Writing self-reflection paper	Example study about existing nursing process application Writing self-reflection paper
	Conference	Day 5	Conference using simulation video	Case study conference
Week 2	Theory-oriented clinical reasoning	Day 6	Deduction clues related to acute myocardial infarction problem Electromyography and arrhythmia learning	Subject disease condition and severity grasping
		Day 7	Deduced clue discussion and subject condition OPT model work sheet presentation	Subject handover Quiz test Selection of a case study with high severity and presentation Nursing problem clue deduction of case study
	Clinical reasoning training	Day 8	Free practice: cardiovascular condition video watching Self-quiz test Free practice: OSCE	Selection of example study subject Free practice: core nursing skill
		Day 9	Simulation training using OPT model Handover using SBAR Writing self-reflection paper	Example study about existing nursing process application Writing self-reflection paper
	Conference	Day 10	Conference using simulation video	Example study conference
Post survey			Clinical reasoning, problem-solving process, self-efficacy, clinical competency	

**Table 2:** Homogeneity in general characteristics of subjects between experimental and control groups (*n* = 45).

Characteristics		Experimental Group (*n* = 25)	Control Group (*n* = 20)	Chi-Square	*p*-Value
		*n* (%)			
Gender	Male	8 (32.0)	3 (15.0)	1.74	0.297
	Female	17 (68.0)	17 (85.0)		
Satisfaction of clinical practicum	Good	16 (64.0)	18 (90.0)	4.07	0.079
	Fair	9 (36.0)	2 (10.0)		
Satisfaction of nursing	Good	13 (52.0)	10 (50.0)	3.76	0.164
	Fair	12 (48.0)	7 (35.0)		
	Poor	0 (0)	3 (15.0)		
Satisfaction of college	Good	9 (36.0)	10 (50.0)	1.68	0.422
	Fair	10 (40.0)	8 (40.0)		
	Poor	6 (24.0)	2 (10.0)		
Grade point average of clinical practicum	A+∼A	13 (52.0)	17 (85.0)	5.45	0.027 *
	B+∼B	12 (48.0)	3 (15.0)		

* *p* < 0.05; Fisher’s exact test.

**Table 3:** Homogeneity for dependent variables of subjects between experimental and control groups (*n* = 45).

Variables	Experimental Group (*n* = 25)	Control Group (*n* = 20)	t-Value	*p*-Value
	Mean ± Standard Deviation			
Clinical reasoning	12.57 ± 11.22	12.23 ± 12.26	−0.58	0.566
Problem-solving process	74.32 ± 17.30	84.85 ± 17.90	−2.00	0.052
Self-efficacy	102.28 ± 29.98	121.45 ± 18.42	−2.50	0.016 *

* *p* < 0.05; paired *t*-test.

**Table 4:** Analysis of covariance of clinical reasoning between experimental and control groups (*n* = 45).

Group	Pretest	Posttest	Source	F-Value	*p*-Value
	Mean ± Standard Deviation				
Experimental group (*n* = 25)	12.32 ± 9.13	32.04 ± 16.53	Group	10.59	0.002 **
Control group (*n* = 20)	14.25 ± 13.24	18.30 ± 12.87	Grade point average of clinical practicum	0.77	0.387

** *p* < 0.01; analysis of covariance.

**Table 5:** Analysis of covariance of problem-solving process between experimental and control groups (*n* = 45).

Group	Pretest	Posttest	Source	F-Value	*p*-Value
	Mean ± Standard Deviation				
Experimental group (*n* = 25)	74.32 ± 17.30	99.92 ± 11.42	Group	30.92	<0.001 ***
Control group (*n* = 20)	84.85 ± 17.90	76.65 ± 17.54	Grade point average of clinical practicum	1.87	0.179

*** *p* < 0.001; analysis of covariance.

**Table 6:** Analysis of covariance of self-efficacy between experimental and control groups (*n* = 45).

Group	Pretest	Posttest		Source	F-Value	*p*-Value
	Mean ± Standard Deviation		Mean Adjusted			
Experimental group (*n* = 25)	102.28 ± 29.98	137.40 ± 22.24	144.15	Group	36.03	<0.001 ***
				Pretest	19.99	<0.001 ***
Control group (*n* = 20)	121.45 ± 18.42	112.45 ± 27.15	104.02	Grade point average of clinical practicum	5.39	0.025 *

* *p* < 0.05, *** *p* < 0.001; analysis of covariance.

**Table 7:** Analysis of covariance of clinical competency between experimental and control groups (*n* = 45).

Group	Posttest	Source	F-Value	*p*-Value
	Mean ± Standard Deviation			
Experimental group (*n* = 25)	31.88 ± 4.46	Group	11.07	0.002 **
Control group (*n* = 20)	18.90 ± 7.15	Grade point average of clinical practicum	0.50	0.484

** *p* < 0.01; analysis of covariance.

## Data Availability

The data presented in this study are available on request to the authors.
